# Follow-up of women with atypical squamous cells cannot exclude high-grade squamous intraepithelial lesions (ASC-H): Acompanhamento de mulheres com células escamosas atípicas não pode excluir lesão intraepitelial de alto grau (ASC-H)

**DOI:** 10.1590/1516-3180.2014.1321597

**Published:** 2014-02-01

**Authors:** Fanny López-Alegría, Dino Soares De Lorenzi, Orlando Poblete Quezada

**Affiliations:** I PhD. Professor, Department of Nursing, School of Nursing, Universidad Andres Bello, Santiago, Chile; II MD. Professor, Department of Obstetrics and Gynecology, Universidade de Caxias do Sul, Rio Grande do Sul, Brazil; III Medical Technologist. Cytology Laboratory, Complejo Asistencial Barros Luco, Santiago, Chile

**Keywords:** Neoplasms squamous cell, Uterine cervical neoplasms, Vaginal smears, Biopsy, Follow-up studies, Neoplasia de células escamosas, Neoplasias do colo do útero, Esfregaço vaginal, Biópsia, Seguimentos

## Abstract

**CONTEXT AND OBJECTIVE:**

The concept that the presence of atypical squamous cells cannot exclude high-grade squamous intraepithelial lesions (ASC-H) was introduced in the 2001 Bethesda System of cervical cytology classification. This nomenclature defines cervical cancer precursor lesions. The objective of this study was to investigate the colpocytological-histological results from a three-year follow-up conducted on a cohort of women with reports of ASC-H who were attended during 2005-2006 at clinics of the Southern Metropolitan Healthcare Service of Santiago, Chile.

**DESIGN AND SETTING:**

Prospective cohort study at primary healthcare clinics in Santiago, Chile.

**METHODS:**

: Colpocytological-histological follow-up was conducted over a three-year period on 92 women with cytological reports of ASC-H who were attended at primary healthcare clinics during 2005-2006.

**RESULTS:**

: At the end of the follow-up period, high-grade lesions were evaluated and the following outcomes were observed: seven women presented invasive cancer (7.6%), 49 presented high-grade lesions (53.3%), 26 presented low-grade lesions (28.2%) and 10 presented normal results (10.9%). The "Conditional Probabilities Tree Diagram" was used to show the results from tests and the times of lesion detection. It demonstrated that, after a first report of ASC-H, clinical management needed to be interventionist.

**CONCLUSION:**

The follow-up on our cohort of women showed that the majority of uncertain ASC-H diagnoses (82.6%) had abnormal colposcopic results and that during the follow-up using ASC-H smears, two out of every three women developed high-grade lesions.

## Abstract (pt)

**CONTEXTO E OBJETIVO:**

O conceito de que à presença de células escamosas atípicas não se pode excluir lesão intraepitelial de alto grau (ASC-H) foi introduzido pelo Sistema de Bethesda 2001, na classificação de citologia cervical. Esta nomenclatura define lesões precursoras do câncer cervical. O objetivo deste estudo foi investigar os resultados colpo-cito-histológicos de três anos de acompanhamento realizado em uma coorte de mulheres com relatórios de ASC-H que receberam atendimento no período 2005-2006 em clínicas do Serviço Metropolitano de Saúde Sul de Santiago, Chile.

**TIPO DE ESTUDO E LOCAL:**

Estudo de coorte prospectivo em unidades básicas de saúde de Santiago, Chile.

**MÉTODOS:**

Foi conduzido um acompanhamento colpo-cito-histológico por um período de três anos em 92 mulheres com laudos citológicos de ASC-H, que receberam atendimento nas unidades básicas de saúde de 2005-2006.

**RESULTADOS:**

No final do período de acompanhamento, as lesões de alto grau foram avaliadas e os resultados foram observados: sete mulheres apresentaram câncer invasivo (7,6%), 49 apresentaram lesões de alto grau (53,3%), 26 apresentaram lesões de baixo grau (28,2%) e 10 apresentaram resultados normais (10,9%). O "Diagrama de Árvore Condicional de Probabilidades" foi utilizado para mostrar os resultados dos testes e o período de detecção das lesões, demonstrando que, depois de um primeiro relatório de ASC-H, o manejo clínico deve ser intervencionista.

**CONCLUSÃO:**

O acompanhamento de nossa coorte de mulheres mostra que a maioria dos diagnósticos incertos de ASC-H (82,6%) tiveram resultado colposcópico anormal e, durante o acompanhamento de esses esfregaços ASC-H, duas de cada três mulheres desenvolvem lesões de alto grau.

## INTRODUCTION

The concept that the presence of atypical squamous cells cannot exclude high-grade squamous intraepithelial lesion (ASC-H) was introduced by the 2001 Bethesda System of cervical cytology classification. This system addresses the need to define a cytological category between atypical squamous cells of undetermined significance (ASCUS) and high-grade squamous intraepithelial lesion (HSIL).^1^ The definition of this nomenclature resulted from the ASCUS/LSIL Triage Study (ALTS) carried out between 1996 and 2000, in which interpretations of ASCUS were subcategorized as "equivocal LSIL" (ASCUS-L) and "equivocal HSIL" (ASCUS-H). Through a consensus among expert pathologists, it was concluded that ASCUS-H represented a cytological category that differed from ASCUS-L and HSIL.^2^ ASCUS-H has been seen to present a risk association of 27.2% with cervical cancer precursor lesions, which is higher than the risk association for the ASCUS-L category of 11.4%, but lower than the risk association for HSIL of 44.8%.^2^ ^,^ ^3^

Subsequent studies, such as a study by Sherman et al., detected high-grade lesions (HSIL) in 41% of the women with initial ASC-H smears.^4^ Supporting previous findings, Patton obtained a high positive predictive value for lesions of cervical intraepithelial neoplasia (CIN II/III), derived from the ASC-H diagnosis category in a general population of women.^5^

Bonvicino et al. used computed data from two medical centers in San Antonio, Texas, United States, in which 260 women diagnosed with ASC-H underwent cytological-histological follow-up, and found that 25.4% had a high-grade lesion at the end of the follow-up period.^6^ Using the same methodology in Brazil, Cytryn et al. found high prevalence of high-grade lesions among a total of 57 ASC-H cases.^7^ Thus, the abovementioned follow-up studies demonstrated that there are connections between ASC-H smears and high-grade lesions.^4^ ^,^ ^6^ ^,^ ^7^

In Chile, the 2001 Bethesda System was adopted in 2005 via Ordinance Number B232 1771 issued by the Department of Non-Communicable Diseases, within the Division of Disease Prevention and Control. This ordinance standardized the use of the nomenclature that had started to be used in previous years.^8^ In 2006, the National Program for Research and Control of Cervical Cancer collected 779,068 Pap smears nationwide, from which 14,608 (1.9%) were found to be atypical using the new nomenclature.^9^

Although other studies such as Gaete et al.^10^ and Yazigi et al.^11^ were conducted in Chile, they were limited because they did not discriminate the type of atypia given that they were conducted using cytological reports that were produced before the 2001 Bethesda System was incorporated in Chile. The scarce scientific evidence regarding the ASC-H category among the female population of Chile motivated the present study.

## OBJECTIVE

The objective of this study was to investigate the cytological-histological results from a three-year follow-up conducted on a cohort of women with reports of ASC-H who were attended during 2005-2006 at clinics within the Southern Metropolitan Healthcare Service in Santiago, Chile. 

## METHODS

The present research comprises a prospective cohort study on women who were followed up over a three-year period. During 2005-2006, 88,438 cervical cytological smears were collected by midwives using Ayre spatulas and cytobrushes in primary healthcare clinics, in the southern area of Santiago, Chile. 

These smear screenings were fixed and sent to Barros Luco Hospital Laboratory (central hospital). In this laboratory, the cytological smears were stained by means of the conventional Pap smear technique and were classified using the national nomenclature system, which is equivalent to the 2001 Bethesda system.^8^ These tests were processed by a team of cytotechnologists with an average of 20 years of experience. Subsequently, these records were registered in the "Cytological Expert Diagnosis Archive System" at the Chilean Ministry of Health. This provided the cytological-histological database that we searched to obtain data for the present research study.^9^

The ASC-H cases were selected (n = 106) and the following selection criteria were applied: the women needed to be without uterine pathological conditions, without prior cervical procedures and with normal Pap smear results for the last three years. These criteria resulted in a cohort of 92 women who were followed up colpocytologically-histologically for a three-year period or until their cases were resolved.

The clinical management of these women included gynecological examinations, cytological tests (Pap smears), colposcopic examinations and biopsies. HPV testing was not performed due to cost constraints. All of the follow-up tests were performed by professionals at the central hospital's Cervical Pathology Clinic.

The variables studied included the following: the women's age at the time of ASC-H identification, the number and type of cytological, colposcopic and histological results and the length of follow-up (months) among the women with ASC-H cytology, which ended with diagnostic confirmation, defined as diagnosis of a more severe lesion (CIN II+) or negative confirmation.

To define the cytological variable (Papanicolaou), the 2001 Bethesda nomenclature was utilized. The results were classified as follows: negative for intraepithelial lesion or malignancy (Neg); atypical squamous cells of undetermined significance (ASCUS); atypical squamous cells that cannot exclude high-grade squamous intraepithelial lesion (ASC-H); low-grade squamous intraepithelial lesion (LSIL); high-grade squamous intraepithelial lesion (HSIL); or squamous cell carcinoma. 

To define the histological variable (biopsy), cervical intraepithelial neoplasia (CIN) grades were used and divided as follows: cervical intraepithelial neoplasia of low grade (CIN I); cervical intraepithelial neoplasia of moderate grade (CIN II); cervical intraepithelial neoplasia of high grade (CIN III); carcinoma *in situ *(CIS); or invasive carcinoma. 

For the colposcopic variable, a standard protocol was used, which included conventional visual examination, application of 5% acetic acid and identification of the squamocolumnar junction. This variable was defined as follows: negative (-) colposcopy, when colposcopic findings did not show severe lesions or the need for a biopsy; or positive (+) colposcopy, when colposcopic findings showed lesions of severity that required biopsy.

The data collected were analyzed electronically using Microsoft Excel (version 2007) and the "Conditional Probabilities Tree Diagram," which shows the number, type, outcome and time interval between patient tests. 

## RESULTS

Out of the 88,438 cervical exfoliative smears collected, 752 (0.85%) were atypical Pap smears. The latter contained atypical squamous cells, which were divided into atypical squamous cells of undetermined significance (ASC-US) (619 cytological tests, 0.69%) and atypical squamous cells that cannot exclude high-grade squamous intraepithelial lesion (ASC-H) (106 cytological tests, 0.11%) (Table 1).

**Table 1:**
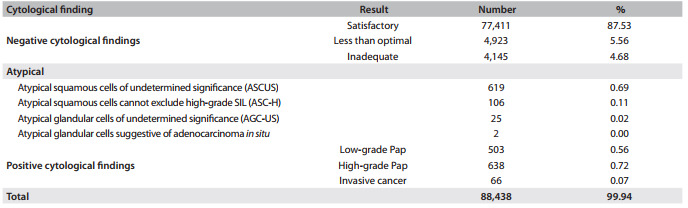
Exfoliative cervical cytological examinations from primary healthcare clinics in Santiago, Chile, 2005 - 2006

After applying the selection criteria to the group of women with ASC-H Pap smears, the cohort consisted of 92 women, with a mean age of 38.2 years and an age range from 19 to 71 years.

**Figure 1:**
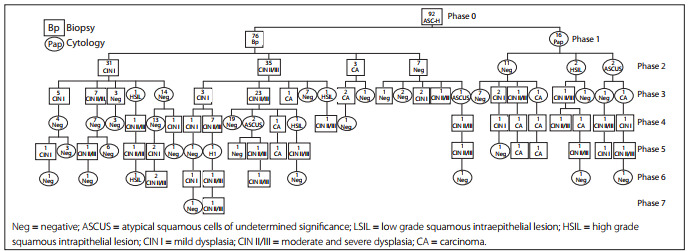
Follow-up of women with high-grade squamous intraepithelial lesions (ASC-H) in Chile.

The follow-up results from this cohort were illustrated using the "Conditional Probabilities Tree Diagram" (Figure 1), which begins with the ASC-H Pap smear ("phase 0") and illustrates the tests performed on the women and their outcomes over time. Squares signify a biopsy and circles indicate a Pap smear. 

It needs to be borne in mind that the ASC-H cytological finding is defined as a suspected case (positive Pap smear) in the cervical cancer program. These 92 women who were attended at primary healthcare clinics were managed in accordance with the central hospital's "management algorithm for specialist or cervical pathology clinic treatment from the time of the first atypical Pap", in order to make the diagnosis and/or proceed with treatment. 

During the first contact with this clinic, the patient's history was taken, a gynecological examination was made and colposcopy was performed. Colposcopy is considered to be the first procedure for diagnostic confirmation in the management algorithm. As shown in "Phase 1", 76 cases (82.6%) showed abnormal colposcopic findings and required biopsy for histological analysis, while in 16 cases (17.4%), the initial colposcopic findings were normal and were followed up by a Pap test. 

The average length of time taken for the patients to complete these medical procedures was 3.15 months, with a minimum of two days and a maximum of 19.6 months.

Subsequently, the cytological-histological results collected in the previous stage were known as "Phase 2". For the group that started with a histological study, the results were distributed as follows: 31 biopsies showed CIN I; 35 showed CIN II/III; three showed carcinoma; and seven were negative. For the group that started with a Pap smear, three of these tests indicated HSIL, 11 were negative for neoplastic cells and two indicated ASC-US. Given these results, and in accordance with the derivation algorithm, a reassessment was performed and the previous procedures were repeated. Pap and colposcopy were obligatory, while the histological examination was made according to the colposcopic findings.

In "Phase 3" and as a result of the repeated examinations, 12 women were found (through biopsy) to have CIN I; 34 women were found to have CIN II/III; three women were found to have carcinoma and four women were negative. In the cases with Pap smears, 38 tests were performed and most of them (33) showed normal outcomes. These results mostly corresponded to patients who had been treated by means of surgical conization consequent to the diagnostic-treatment biopsies that were performed prior to gathering material for Pap smears. Following conization, and in accordance with the Chilean Ministry of Health's guidelines, two Pap smears must be performed, with an interval of six months, before the patient returns to the primary healthcare clinic. 

In "Phase 4", on the one hand, the outcomes from the earlier 14 biopsies are shown: four were classified as carcinoma, six as high-risk lesions and four as low-risk lesions. On the other hand, cytological outcomes are shown, and these mostly related to patients according to the process of epidemiological monitoring. 

In "Phase 5," there were also results with high-grade lesions from a total of 20 women. In "Phases 6 and 7", the cases were predominantly monitored by means of Pap smears and there were four patients with CIN II/III and one with CIN I who was diagnosed via biopsy. 

In this diagnostic search process, 166 oncotic cytological tests were performed via conventional Pap smears, with an average of 1.8 examinations per woman and a minimum of zero and maximum of five cytological tests per woman. With regard to histological examinations, the total numbers of uterine cervix biopsies guided by colposcopy was 158, with an average of 1.7 per woman, and a minimum of zero and maximum of four. The total number of cytological-histological examinations that patients required to obtain a definitive diagnosis was as follows:

- 1 biopsy = 27 women (29.3%);
- 2 biopsies = 46 women (50%);
- 3 biopsies = 8 women (8.7%);
- 4 biopsies = 3 women (3.3%);
- 2 Papanicolaou smears = 8 women (8.7%);

The average length of time from the ASC-H Pap smear to the final diagnosis of the most severe lesion was 5.29 months in the biopsy group and 12.26 months in the Pap smear group. In evaluating this three-year cytological-histological follow-up period, the 92 women were found to have the following lesion distribution:

- 7 women had invasive carcinoma (7.6%);
- 49 women had high-grade lesions (53.3%);
- 26 women had low-grade lesions (28.2%);
- 10 women had normal cytological outcomes (10.9%).

## DISCUSSION

ASC-H is a type of atypical smear characterized by follow-up that includes a range of clinical procedures to reach a definitive diagnosis. Knowledge of the prevalence of women carrying ASC-H is essential for estimating the proportion of women at risk of developing a high-grade lesion. 

One of the first studies aimed at investigating the prevalence of this new cytological classification category was carried out by the College of American Pathologists in the United States in 2002-2003: this was a nationwide survey and it revealed that ASC-H represented approximately 0.2% of cytological interpretations.^12^ These first studies indicated that using this category was relatively congruent with the frequency provided by the 2001 Bethesda System.^1^ Thus, studies including this new category were started worldwide, thereby providing ranges of ASC-H prevalence. This prevalence ranged from 0.22% out of a total of 27,367 Pap smears performed in a screening program in India, up 8.8% out of a total of 12,188 Pap smears in South Africa.^13^ ^,^ ^14^

In Latin America, and specifically in Brazil, Yamamoto et al. found ASC-H smear prevalences of 0.23% in 2007 and 0.54% in 2008, out of a total of 56,179 smears collected through the Cervical Cancer Research and Control Program.^15^ Within this line of research, our study results, which demonstrated ASC-H prevalence of 0.11%, or 106 Pap smears out of a total of 88,438 between 2005 and 2006, are congruent with the established 2001 Bethesda System (Table 2).^5^ ^,^ ^13^ ^-^ ^24^

**Table 2:**
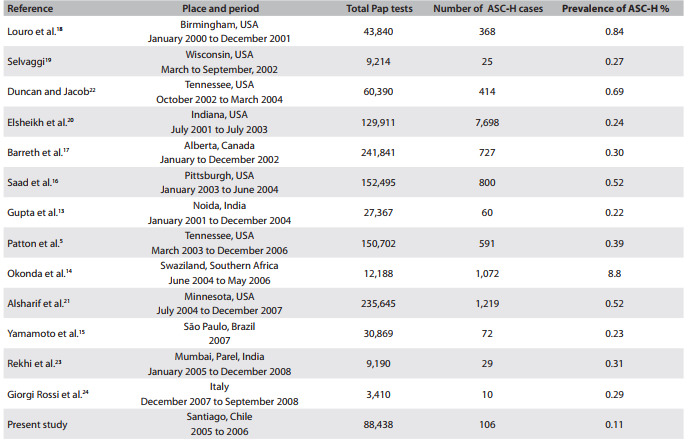
Prevalence of atypical squamous cells that cannot exclude high-grade squamous intraepithelial lesions (ASC-H) in population screening studies

Concerning the percentage of ASC-H among the total volume of atypical squamous cells (ASC), the 2001 Bethesda System indicates that between 5 and 10% (approximately) should be expected.^1^ ^,^ ^3^ In our study, the ASC-H prevalence (106) represented 14% of the total atypia (752 women). The national survey conducted by the College of American Pathologists (2002-2003) found that 5% of the squamous atypias corresponded to ASC-H.^12^ In Brazil, Yamamoto et al. obtained 2,622 atypical Pap smears in 2007-2008 and 210 (0.8%) of these were ASC-H smears.^15^

For the clinical management of patients with ASC-H smears, the American Society for Colposcopy and Cervical Pathology (ASCCP) published an algorithm in relation to clinical management of these patients in order to unify the criteria.^25^ In Chile, and taking this algorithm into consideration, the Chilean Ministry of Health implemented its "Clinical Guidance for Cervical Cancer" (2005 and update 2010), which includes flow charts for clinical decision-making, such as the "management algorithm for specialists or Cervical Pathology Clinics (CPC) from the time of the first atypical Pap smear according to the 2001 Bethesda System" and the "algorithm of diagnostic confirmation". In the first algorithm, it is established that women with atypical smears must be managed at a cervical pathology clinic.^26^ ^,^ ^27^ The follow-up for our population study was started at a cervical pathology clinic. The population's demographic profile comprised young women (average age: 38.2 years), which was similar to the populations of several other studies, which had averages ranging from 29 to 38.1 years. It is noteworthy that these outcomes corresponded to women in the screened population (Table 3).^6^ ^,^ ^13^ ^,^ ^17^ ^,^ ^18^ ^,^ ^28^

**Table 3:**
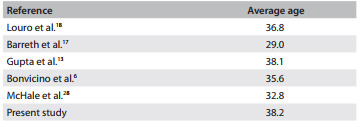
Average age of women with atypical squamous cells that cannot exclude high-grade squamous intraepithelial lesions (ASC-H) in population screening studies

In the present study, women with ASC-H atypia underwent on average 1.8 cytological tests per woman (0 to 5 Pap smears) and half of them (46 women) underwent a minimum of two biopsies, in order to reach the definite CIN II+ diagnosis. It is possible to compare these outcomes with Bonvicino's data, in which among 260 ASC-H smears, there was an average cytological-histological follow-up of 1.35 Pap smears per woman, with a range of 1 to 4, and the biopsy follow-up consisted of an average of 0.64 biopsies per woman. In other words, the majority of the women (72%) underwent only one biopsy for a diagnosis of CIN II+ to be reached.^6^

The average length of follow-up in the present study was 5.29 months, to reach the most severe diagnosis (CIN II+), using only the biopsy. For confirmation of this cytological-histological diagnosis, a period of 12.26 months was needed, during which two normal cytological examinations were needed, with an interval of six months between them. These results are contrary to those of Bonvicino, in which the CIN II/III diagnosis required 18.5 months.^6^

In our study, 60.9% (56 women) had a definitive high-grade diagnosis (CIN II+) that was verified by biopsy. Comparing this ratio with the literature, we found that it was intermediate between the studies by Saad et al.^16^ and Barreth et al.,^17^ which found that 6% and 79.9% of the women had high-grade lesions, respectively. Table 4 shows 14 studies in which different variables were analyzed. For instance, Louro et al.^18^ made a comparison between conventional collection and liquid-based smears, and found that in spite of being different techniques for cytological sample collection, they resulted in similar definitive diagnoses of CIN II.^18^ This is consistent with the results obtained from the majority of the studies observed, which demonstrated CIN II+ prevalences of more than 40%, according to the biopsy results.^3^ ^,^ ^5^ ^,^ ^6^ ^,^ ^17^ ^-^ ^24^ ^,^ ^29^

**Table 4:**
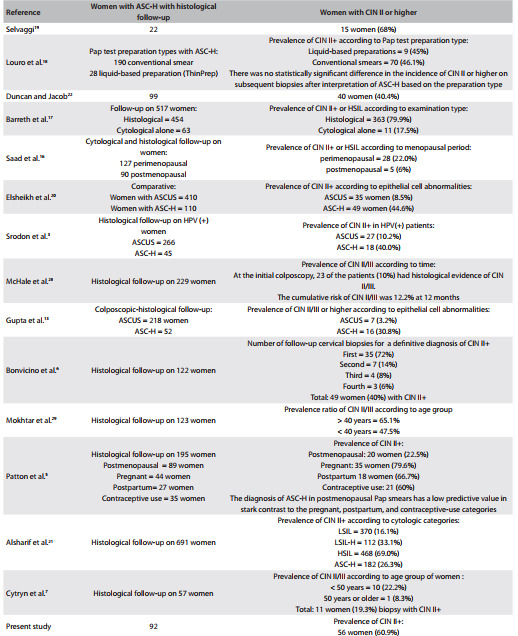
Outcomes from histology follow-up of women with smears showing atypical squamous cells that cannot exclude high-grade squamous intraepithelial lesions (ASC-H)

CIN = cervical intraepithelial neoplasia; LSIL = low grade squamous intraepithelial lesion; HSIL = high grade squamous intrapithelial lesion.

Our results confirmed that at the time of the first ASC-H report, immediate intervention with colposcopy was necessary, followed by a biopsy. This validates the medical management of ASC-H that is required by the "management algorithm from the time of the first atypical Pap," which is included in the Chilean Ministry of Health's Clinical Guidelines for Cervical Cancer.

## CONCLUSIONS

The follow-up of our cohort of women showed the following:

- The majority of the uncertain ASC-H diagnoses (82.6%) also had abnormal colposcopic results.
- During the follow-up on ASC-H smears, two out of every three women developed high-grade lesions (CIN II+), which were detected after an average of 3.15 months.
- Detection of high-grade lesions in a female population through screening reflects the importance of public health programs within the National Cervical Cancer Research and Control Program in Chile.
